# Machine learning analysis of motor evoked potential time series to predict disability progression in multiple sclerosis

**DOI:** 10.1186/s12883-020-01672-w

**Published:** 2020-03-21

**Authors:** Jan Yperman, Thijs Becker, Dirk Valkenborg, Veronica Popescu, Niels Hellings, Bart Van Wijmeersch, Liesbet M. Peeters

**Affiliations:** 1 Theoretical Physics, Hasselt University, Diepenbeek, Belgium; 2 I-Biostat, Data Science Institute, Hasselt University,, Diepenbeek, Belgium; 3 Department of Immunology, Biomedical Research Institute, Hasselt University, Diepenbeek, 3590 Belgium; 4 Rehabilitation and MS-Center, Pelt, 3900 Belgium; 5 REVAL Rehabilitation Research Center, BIOMED, Faculty of Rehabilitation Sciences, Hasselt University, Hasselt, Belgium

**Keywords:** Evoked potentials, Multiple sclerosis, Machine learning, Disease prognosis, Feature extraction

## Abstract

**Background:**

Evoked potentials (EPs) are a measure of the conductivity of the central nervous system. They are used to monitor disease progression of multiple sclerosis patients. Previous studies only extracted a few variables from the EPs, which are often further condensed into a single variable: the EP score. We perform a machine learning analysis of motor EP that uses the whole time series, instead of a few variables, to predict disability progression after two years. Obtaining realistic performance estimates of this task has been difficult because of small data set sizes. We recently extracted a dataset of EPs from the Rehabiliation & MS Center in Overpelt, Belgium. Our data set is large enough to obtain, for the first time, a performance estimate on an independent test set containing different patients.

**Methods:**

We extracted a large number of time series features from the motor EPs with the highly comparative time series analysis software package. Mutual information with the target and the Boruta method are used to find features which contain information not included in the features studied in the literature. We use random forests (RF) and logistic regression (LR) classifiers to predict disability progression after two years. Statistical significance of the performance increase when adding extra features is checked.

**Results:**

Including extra time series features in motor EPs leads to a statistically significant improvement compared to using only the known features, although the effect is limited in magnitude (*Δ*AUC = 0.02 for RF and *Δ*AUC = 0.05 for LR). RF with extra time series features obtains the best performance (AUC = 0.75±0.07 (mean and standard deviation)), which is good considering the limited number of biomarkers in the model. RF (a nonlinear classifier) outperforms LR (a linear classifier).

**Conclusions:**

Using machine learning methods on EPs shows promising predictive performance. Using additional EP time series features beyond those already in use leads to a modest increase in performance. Larger datasets, preferably multi-center, are needed for further research. Given a large enough dataset, these models may be used to support clinicians in their decision making process regarding future treatment.

## Background

Multiple sclerosis (MS) is an incurable chronic disease of the central nervous system (CNS). Because of inflammation there is demyelination and degeneration of the CNS, and patients acquire symptoms which depend on the site of the lesions. Typical MS symptoms include sensation deficits and motor, autonomic and neurocognitive dysfunction. The clinical course of MS can span decades, and varies greatly between individuals [1]. Although there is currently no cure, there are numerous disease-modifying treatments (DMTs) that alter the natural disease course, with more on the way [2].

For the time being, it remains impossible to accurately predict the disease course of an individual patient. This unpredictability causes anxiety and frustration for patients, families and health-care professionals [3]. Ideally, MS should be featured by an individualized clinical follow-up and treatment strategy. A fast and sensitive detection of non-response to the current treatment could trigger a treatment switch, which would optimize the individual treatment trajectory. While there are numerous biomarkers available for MS, there is still discussion on their relative usefulness. Besides magnetic resonance imaging (MRI) scans, which visualize lesions in the CNS, other clinical parameters such as the expanded disability status scale (EDSS) [4] are used in the assessment of MS disease progression [5–9]. Several research groups have shown that evoked potentials (EP) allow monitoring of MS disability and prediction of disability progression [10–32], see [33, 34] for reviews. However, the precise value of EPs as a biomarker for monitoring MS is still under discussion [35–37]. EP provide quantitative information on the functional integrity of well-defined pathways of the central nervous system, and reveal early infra-clinical lesions. They are able to detect the reduction in electrical conduction caused by damage (demyelination) along these pathways even when the change is too subtle to be noticed by the person or to translate into clinical symptoms. EP measure the electrical activity of the brain in response to stimulation of specific nerve pathways or, conversely, the electrical activity in specific nerve pathways in response to stimulation of the brain. Different types of EP are available corresponding to different parts of the nervous system [38]. For visual EP (VEP) the visual system is excited and conductivity is measured in the optic nerve; for motor EP (MEP) the motor cortex is excited and conductivity is measured in the feet or hands; for somatosensory EP (SEP) the somatosensory system (touch) is excited and conductivity is measured in the brain; and for brainstem auditory EP (BAEP) the auditory system (ears) is excited and conductivity is measured at the auditory cortex. If several types of EP are available for the same patient this is referred to as a multimodal EP (mmEP).

Considerable community effort has been performed to summarize mmEP by a one-dimensional statistic, called the EP score (EPS), by applying different scoring methods [14, 15, 18, 19, 23, 31, 32]. The scoring methods described in literature use a limited number of features from these EP time series (EPTS). The latency (i.e. the time for the signal to arrive) is always included. Besides latency, amplitude and dispersion pattern are also possibly included in the EPS [23]. By only using two or three variables extracted from the EPTS, possibly useful information is lost. In this study, we investigate whether a machine learning approach that includes extra features from the EPTS can increase the predictive performance of EP in MS.

Predicting disability progression is often translated to a binary problem, where a certain increase in EDSS is considered as a deteriorated patient. In the literature, the main modeling techniques are linear correlation of latency or EPS with EDSS, and linear or logistic regression models. Except for one study with 30 patients [13], no study has used an independent test set to asses model performance. Some studies use cross-validation to estimate model performance [17, 19, 20, 22, 27]. Akaike information criterion (AIC) or Bayesian information criterion (BIC) are sometimes included to encourage model parsimony. While such models are statistically rigorous, insightful, and often used in practice [39], a realistic performance estimate is obtained by training on a large dataset (part of which is used as a validation set to tweak any hyperparameters), and testing on an independent large dataset containing different patients. This study provides, for the first time, such a performance estimate.

We recently extracted a large number of EPTS from the Rehabilitation & MS Center in Overpelt, Belgium. This patient cohort consists of individuals undergoing treatment. This is the most relevant scenario, since in a clinical setting the majority of patients will have had some form of treatment prior to these types of measurements. The resulting dataset, containing the full time series of mmEP with longitudinal information for most patients, is the first of its kind. We perform a disability prediction analysis on the MEP from this dataset, as this EP modality is most abundant in the dataset. A machine learning approach is used to see if there is extra information in the MEP for predicting disability progression after 2 years, besides latency and amplitude. 419 patients have at least one measurement point, where previous studies had between 22 and 221 patients. Including extra EP features leads to a statistically significant increase in performance in predicting disability progression, although the absolute effect is small. Our results suggest that this effect will become more stable on a larger dataset. We show that a nonlinear model (random forests) achieves significantly better performance compared to a linear one (logistic regression).

The best model for predicting disability progression after 2 years achieves an average area under the curve (AUC) of the receiver-operating characteristic (ROC) of 0.75. In the literature, AUC ROC values for this task range from 0.74 to 0.89, with prediction windows between 6 months and 20 years [20, 23, 24, 26, 27]. The predictive performance of the MEP in this work, achieved on an independent test set and measured in a real-world setting, shows MEP can be a valuable biomarker in clinical practice for disease monitoring and the prediction of disability progression. If our identified extra features are confirmed in larger, multi-center studies, they can be used to give additional feedback to the caregivers on the disease evolution.

## Methods

### Measurement protocol

**Fig. 1:**
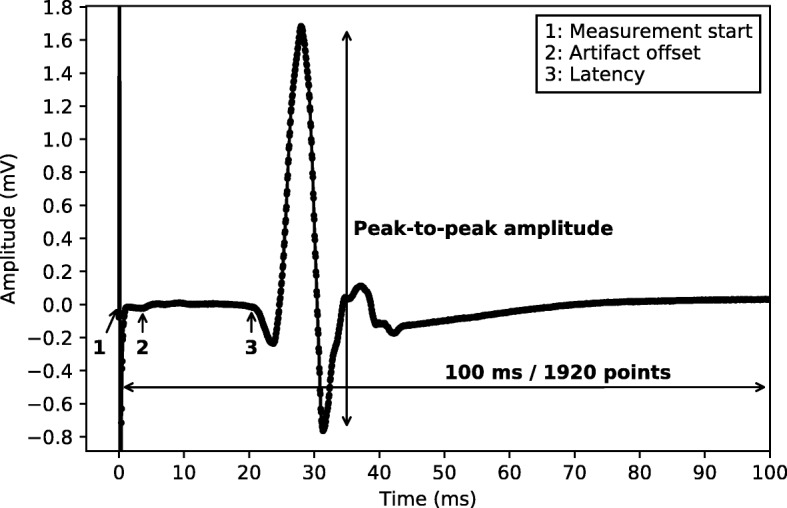
Single MEP An example of a single motor evoked potential (MEP) measurement. The annotations indicate the following points: **1:** The point where the measurement starts, i.e., the moment the motor cortex is stimulated. **2:** As the first 70 points of the measurement often contain artifacts we discard all points up to this point. **3:** The latency of the signal, as annotated by specialized nurses. The time series consists of 1920 values

Motor evoked potentials were recorded from the preinnervated abductor pollicis brevis (APB) and abductor hallucis (AH) muscles bilaterally. Magnetic stimuli were delivered to the hand and leg areas of the motor cortex with a Magstim 2002 or Bistim device (The Magstim Company Ltd., Whitland, UK) via a round coil with an inner diameter of 9 cm with maximal output of the stimulator (2.2 T). Recording is done with two different machines. The signal is recorded for 100ms, starting from the moment the stimulus is applied. The resulting signal is digitized at a frequency rate of 20 kHz or 19.2 kHz (depending on which machine was used), resulting in 2000 or 1920 data points per measurement respectively. One such measurement is illustrated in Fig. 1. The 20 kHz signals are down-sampled to 19.2 kHz. Signals from one machine are filtered between 0.6 Hz and 10 kHz, while the other machine has a high-pass filter of 100 Hz.

For the hands, electrodes are placed at three places: on top of the hand (ground), the APB muscle, and the proximal phalanx of the thumb. The first excitation is at 45% of the maximal stimulator output. New stimuli are presented with an increase of 5 percentage points. The measurement ends if the amplitude reaches 1 millivolt, or if the amplitude stops increasing for stronger stimuli. If the signal is of bad quality, as judged by the nurse, it is discarded.

For the feet, electrodes are placed at three places: on top of the foot (ground), the big toe, and the AH muscle. The first excitation is at 50% of the maximal stimulater output. New stimuli are presented with an increase of 5 percentage points. The measurements end if the amplitude reaches 1 millivolt, or if the amplitude stops increasing for stronger stimuli. If the signal is of bad quality, as judged by the nurse, it is discarded.

**Fig. 2:**
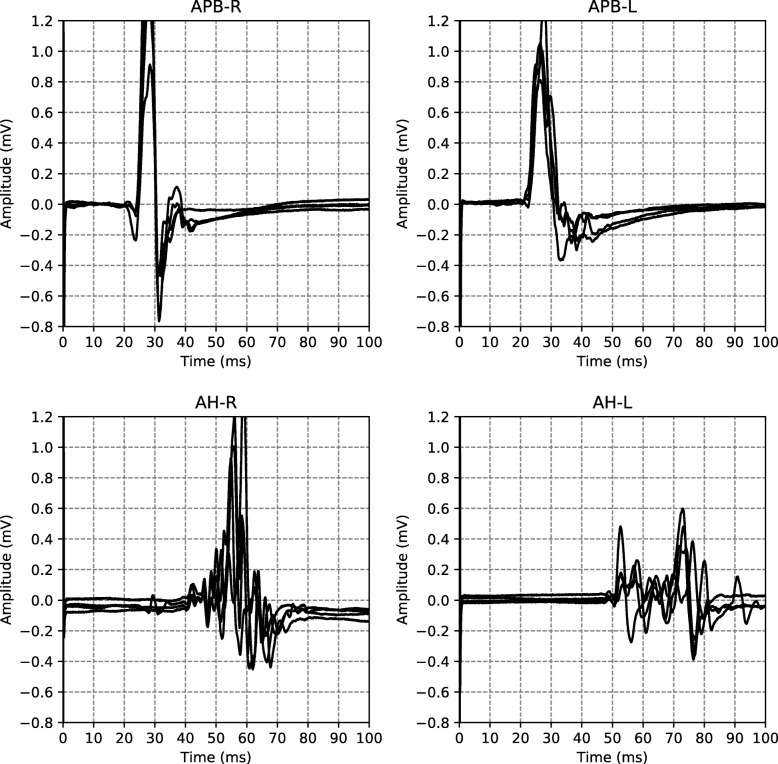
EPTS example Example of the EPTS measured during a single patient visit. The titles indicate the anatomy (APB for the hands, AH for the feet) and the respective sides (L for left, R for right)

An example of all the EPTS of the MEP for a single visit is shown in Fig. 2. For each limb, each excitation strength gives one EPTS. After discussion with the neurologists we decided to use only the EPTS with the maximal peak-to-peak amplitude, as this is likely to be the most informative measurement.

### Dataset

The full evoked potential dataset consists of 642 patients and has SEP (528), BAEP (1526), VEP (2482), and MEP (6219) visits (dataset paper to be published). We only study the MEP, because they are most frequently measured. Each MEP visit contains 4 measurements: two for the hands (APB muscle), and two for the feet (AH muscle). Visits that don’t contain all 4 EPTS are discarded.

We use the standard definition of disability progression [40], where the patient has progressed if $\text {EDSS}_{T_{1}} - \text {EDSS}_{T_{0}} >= 1.0$ for $\text {EDSS}_{T_{0}} \leq 5.5$, or if $\text {EDSS}_{T_{1}} - \text {EDSS}_{T_{0}} >= 0.5$ for $\text {EDSS}_{T_{0}} > 5.5$. T_0_ is the time of the first measurement, and T_1_ is the time of the EDSS measurement between 1.5 and 3 years which is closest to the 2 year mark. The MEP visit has to occur 1 year before or after T_0_. Visits without two-year follow-up are discarded. We do not perform confirmation of disability progression, where one confirms progression with one or a few EDSS measurements after $\text {EDSS}_{T_{1}}$ [40]. This makes it more straightforward to compare our results with the literature on predicting disability progression from EPs, where this is also not done. It furthermore gives us more input-output pairs for training the model, where the input is a collection of measurement variables (e.g. latency, peak-to-peak amplitude, age), and the output is the disability progression target (yes or no). From now on we refer to input-output pairs as samples. The downside is that our target is likely more noisy, as some positive targets are not truly progressed in disability, but rather fluctuations in the measurement or disease process.

Measurements of a duration differing from 100ms are discarded. Around 97% of the data has duration 100ms, so this has little influence on the dataset size, and keeps the data homogeneous. The majority of EPTS consist of 1920 data points. Due to a slight difference in the sampling rate (cfr. “Measurement protocol” section), some EPTS consist of 2000 data points. These EPTS were downsampled to 1920 data points.

The latencies (as illustrated in Fig. 1) were manually annotated by specialized nurses during routine clinical follow-up, and are included in the extracted dataset. Their test-retest reliability has not been calculated for this dataset, but the literature indicates that they are reliable [41–43], and can be used as a biomarker to study MS disease progression [44]. The peak-to-peak amplitude was calculated by taking the difference between the minimum and maximum value of the whole EPTS.

In case no spontaneous response or MEP in rest position is obtainable, a light voluntary contraction of the muscle in question is asked in order to activate the motor cortex and increase the possibility of becoming a motor answer. This so called facilitation method is usually very noisy due to baseline contraction of the muscle measured, so we decided to drop them from the dataset altogether. Facilitated measurements are characterized by a non-flat signal right from the start of the measurement. We drop any EPTS that have a spectral power above an empirically determined threshold at the starting segment of the measurement. This segment is determined by the values of the latency of a healthy patient, which we set to be 17 ms as this is the lower bound for the hands. We use the same threshold for the feet, which is not a problem since the lower bound there is higher.

The type of MS was inferred from the diagnosis date and the date of onset, both of which have missing values, making the type of MS field somewhat unreliable.

After all these steps, we are left with a dataset of 10 008 EPTS from 2502 visits of 419 patients. Note that one patient can have several visits that satisfy the conditions for two-year follow-up. We have one target (worsened after 2 years or not) for each visit, so the total number of samples is 2502. Some of the characteristics of the dataset are summarized in Table 1.

**Table 1:** Characteristics of the dataset

**MS type**	**# patients**	**age (SD)**	**EDSS (SD)**	**F / M ratio**	**# visits**	**% worsening**
Unknown	138	44 (12)	3.1 (2.0)	98 / 40	558	13.1
CIS	7	31 (11)	1.3 (0.8)	7 / 0	9	11.1
PPMS	12	57 (11)	4.0 (1.4)	7 / 5	74	12.2
RRMS	223	44 (11)	2.6 (1.5)	164 / 59	1592	9.6
SPMS	45	56 (8)	5.0 (1.5)	31 / 14	269	14.5
All	419	45 (12)	3.0 (1.8)	301 / 118	2502	11.0

The final column (% worsening) represents the percentage of visits of patients that have worsened 2 years later. Abbreviations used: MS multiple sclerosis, SD standard deviation, EDSS expanded disability status scale, F female, M male, CIS clinically isolated syndrome, PPMS primary progressive MS, RRMS relapsing-remitting MS, SPMS secondary progressive MS

### Data analysis pipeline

We start with a simple model that uses a subset of the features proposed in the literature. As other EPS require neurologist interpretation, and are therefore difficult to automate, we use latencies in our baseline. The fact that this is a fair baseline is supported by [23], where it was shown that different EPS have similar predictive performance, with short-term change or baseline values in (z-scored) latencies being more predictive than changes in other EPS. It is furthermore supported by the results from [17], where the central motor conduction time of the MEP was more informative for disability progression than the MEP EPS.

**Fig. 3:**
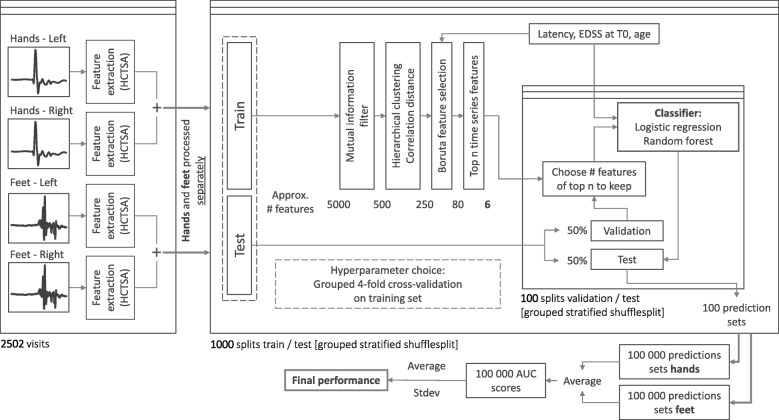
Pipeline Schematic overview of the data analysis pipeline. By grouped stratified shufflesplit we mean splits of train/test sets generated by assigning samples to train or test set at random, but keeping the patients in test and training set separated, and making sure the ratio of positive targets are roughly the same for the test and training set. To assess the impact of the size of the training set, we run this pipeline for 4 different ratios of train/test set size

Despite the increased size of the dataset, the disability progression classification task remains a challenging problem. Challenging aspects are the limited sensitivity to change of the EDSS measure, its dependence on neurologist interpretation, and the heterogeneity of disease development. Therefore, our data analysis pipeline is mainly focused on minimizing overfitting. As our dataset includes the full EPTS, we wish to find one or more time series features that provide supplemental information on disability progression, on top of the features already used in the literature. A schematic overview of the data analysis pipeline is shown in Fig. 3. The various steps in the data analysis pipeline are detailed below. The data analyis pipeline was implemented in Python using the scikit-learn library [45], with the exception of the Boruta processing step, for which we used the Boruta package in R [46], and the feature extraction, for which we used the highly comparative time-series analysis (HCTSA) package [47, 48], which is implemented in Matlab and is available on github (https://github.com/benfulcher/hctsa). To determine sensible values for the hyperparameters of the model, we performed grouped 4-fold cross-validation on the training set. For more details on this, along with an analysis of the robustness of the performance of the model to these choices of hyperparameters, we refer the reader to Additional file 1.

**Feature extraction:** Because each EPTS starts with a large peak at the beginning, an uninformative artifact of the electrophysiological stimulation, the first 70 data points of each EPTS are discarded. A diverse and large set of time series features is extracted from the rest of the EPTS (1850 data points) with the HCTSA package, which automatically calculates around 7700 features from different TS analysis methodologies. The motivation for this approach is to perform a wide variety of time series analysis types, and draw general conclusions on what approaches are useful. It makes the analysis less subjective, since one does not have to choose a priori the type of hand-engineered features that are extracted. Given the large size of this feature set, one expects that almost all useful statistical information contained in the EPTS is encoded in it. A detailed discussion of the HCTSA library and its included features can be found in the manual of its git repository (https://hctsa-users.gitbook.io/hctsa-manual/) and in the supplementary information of [47]. There are several highly performant time series classification libraries available (e.g. [49]). The advantage of using HCTSA is interpretability: after feature selection, the final model contains one to a few extra features, whose content can be investigated. This in contrast to more black-box type classifiers that take the whole signal as input and return an output. The underlying philosophy is very similar to, e.g., radiomics for the analyses of MRI images [50]. To our knowledge, HCTSA is the only library that computes such a comprehensive set of time series features.

The feature matrix **F**_*ij*_ has rows *i* for each EPTS and columns *j* for each feature. If a column **f**_*j*_ contains an error or NaN value it is discarded. Normalization is performed by applying the following transformation on each column:

1 $$ \hat{\mathbf{f}}_{j} = \left\{ 1 + \exp \left[ - \frac{\mathbf{f}_{j} - \text{median}(\mathbf{f}_{j})}{\text{iqr}(\mathbf{f}_{j}) / 1.35} \right] \right\}^{-1},  $$

with iqr the interquartile range. Because the median and iqr are used, this normalization is robust to outliers. All normalized columns $\hat {\mathbf {f}}_{j}$ that contain an error or NaN are discarded. To exploit the symmetry between the measurements performed on the left and the right limb, we sum the features of both sides. This reduces the number of features we need to consider, which is helpful against overfitting. The final normalized feature matrices $\hat {\mathbf {F}}_{ij}$ of AH and APB both have size 5004 ×5885.

**Mutual Information:** Our goal is to use a feature selection algorithm in order to determine the most important features. The ratio of the number of samples to the number of features is quite small (≈ 1). The feature selection algorithm we use, Boruta [46], was expected to work well for such a ratio [51]. We however found it to perform poorly for our problem. The performance of Boruta was tested by adding the latency, which is known to be relevant, to the list of candidates, which was subsequently not marked as relevant by Boruta. We therefore reduce the number of features using mutual information with the target as a measure of feature importance. We select the top ten percent of features based on this metric. We performed an analysis of the impact of this choice of preselection method, for which we refer the reader to Additional file 1.

**Hierarchical clustering:** In this step we seek to reduce redundancy in our choice of features. We estimate this redundancy using the correlation distance, which we define here as

2 $$ \text{correlation distance} = \left|\frac{\left(\mathbf{u} - \bar{\mathbf{u}}\right)\cdot\left(\mathbf{v} - \bar{\mathbf{v}}\right)}{\left\lVert \left(\mathbf{u} - \bar{\mathbf{u}}\right) \right\rVert_{2} \left\lVert \left(\mathbf{v} - \bar{\mathbf{v}}\right) \right\rVert_{2}}\right|  $$

where **u** and **v** are the feature vectors we wish to compare, and ∥·∥_2_ the Euclidean distance. Note that we take the absolute value here so highly anti-correlated features are filtered as well. Features which are highly correlated have a distance close to zero, and conversely features which are not correlated have a distance close to 1. We cluster all features at a cutoff of 0.1 and keep only one feature for each cluster. This step roughly halves the number of features that remained after the mutual information selection step. We performed an analysis of the impact of this step on the final result, for which we refer the reader to Additional file 1.

**Boruta** With the number of features now reduced to a more manageable count we run the Boruta algorithm [46] to estimate the importance of the remaining features. In a nutshell, the Boruta algorithm compares the importance (as determined by a z-scored mean decrease accuracy measure in a random forest) of a given feature with a set of shuffled versions of that feature (called shadow features). If a feature’s importance is significantly higher than the maximal importance of its set of shadow features, it is marked as important. Conversely, any feature with importance significantly lower than the maximal importance of its shadow features is marked as unimportant, and is removed from further iterations. This procedure is repeated until all features are have an importance assigned to them, or until a maximal number of iterations is reached. Because the Boruta method is based on random forests, and because we use random forests as our classifier, we expect it to be well suited for the feature selection task. We add a few literature features to the set of TS features as well (latencies, EDSS at *T*_0_ and age). There are multiple reasons for doing this. First off, it allows us to check the performance of the Boruta algorithm, as these features are known to be important. Secondly, some of the TS features may only be informative in conjunction with a given literature feature. Boruta returns a numerical measure of feature importance, which allows us to assign an ordering to the features. On average, some 80 features are confirmed to be relevant. From these we select the 6 most important ones, based on their importance score. This cutoff was chosen empirically using cross-validation (cfr. Additional file 1), as more features leads to overfitting of the classifier.

**Classifier** For the final classification we use a random forest, with 100 decision trees and balanced class weights. Using more trees led to no improvement in cross-validation (cfr. Additional file 1). We opted for a random forest classifier due to the fact that it is a non-linear classifier, which is known to be robust against overfitting [52]. It is a popular choice for machine learning tasks involving relatively small datasets. We regularize the model further by setting the minimal number of samples required for a split to be 10% of the total number of samples. This value was obtained using cross-validation on the training set (cfr. Additional file 1). The maximum depth of the resulting decision trees averages around 8. As linear models are most often used in the literature, we use logistic regression for comparison. Furthermore, logistic regression is often used as a baseline in these types of machine learning tasks.

As discussed earlier, we have 4 time series per visit, 2 of the hands (left and right), and 2 of the feet (left and right). We run the pipeline for the hands and feet separately and average the predictions of the resulting classifiers to get the final prediction. This approach was chosen for two reasons: The time series resulting from the measurements are quite disparate, therefore the same time series features may not work well for both. The other reason is that adding too many features to the model causes the classifier to overfit. Splitting up the task like this reduces the number of features per model.

We found that the performance of the algorithm is greatly influenced by the choice of training and test set. To get a measure for how much this factors in we run this data analysis pipeline 1000 times, each time with a different choice of train/test split. That way we can get a better understanding of the usefulness of this process, rather than focusing on a single split. It also drives down the standard error on the mean performance estimate, allowing for a more accurate quantification of the performance increase we get by adding additional time series features to the model. We ensure that patients don’t occur both in the training and the test set, and that the balance of the targets is roughly the same for the train and the test set. We refer to this as grouped (by patients) stratified shuffle splits in Fig. 3, the shuffle pertains to the fact that the samples are distributed randomly across the train and test set for each split, subject to the aforementioned conditions. To illustrate, for some splits we actually obtain AUC values of 0.97, whereas others are random at 0.5. Of course, these are just the extreme values, the performance turns out to be normally distributed around the reported results. At this point, we have the ranking of the 10 most important features as determined by the Boruta algorithm. For the final prediction, we will add the top-*n* features. The value of *n* is determined on a validation set (as illustrated in Fig. 3). For each train-test split, we use half of the test set as a validation set. This split into validation and test set satisfies the same conditions as before, and we evaluate the model for 100 such splits. So in total 1000 models are trained (on the training set), and are subsequently evaluated 100 times each, leading to 100 000 test set performances.

There is a trade-off to be taken into account. On the one hand we want as much data as possible to fit our model, which would require allocating as much data as possible to the training set. On the other hand, however, we want to accurately measure the performance of said model on an independent test set, which for a heterogeneous dataset also requires a large amount of data to minimize the variance. To get an idea of both extremes we evaluate the pipeline at various splits of the dataset. We run the entire pipeline for 4 different sizes of the training set, composed of 20, 30, 50 and 80 percent of the dataset. This method also gives information on the necessary dataset size to achieve a certain performance [53, 54] and on how much room for improvement the algorithm has when given more data [55, 56].

## Results

### Disability progression task

Here we present the results of the disability progression prediction task. In the literature, the main features that are considered are: Latency, EDSS at *T*_0_, peak-to-peak amplitude, age, gender and type of MS. We note that not all of these are found to be significant in the literature (see, e.g., [26]). Using cross-validation (cfr. Additional file 1) we determined that using the latencies of the left and right side separately, the EDSS at *T*_0_ and the age worked best for this prediction task. Adding additional literature features leads to a negligible performance increase. We assess the performance of the literature features as well as the performance when we add additional time series features.

**Fig. 4:**
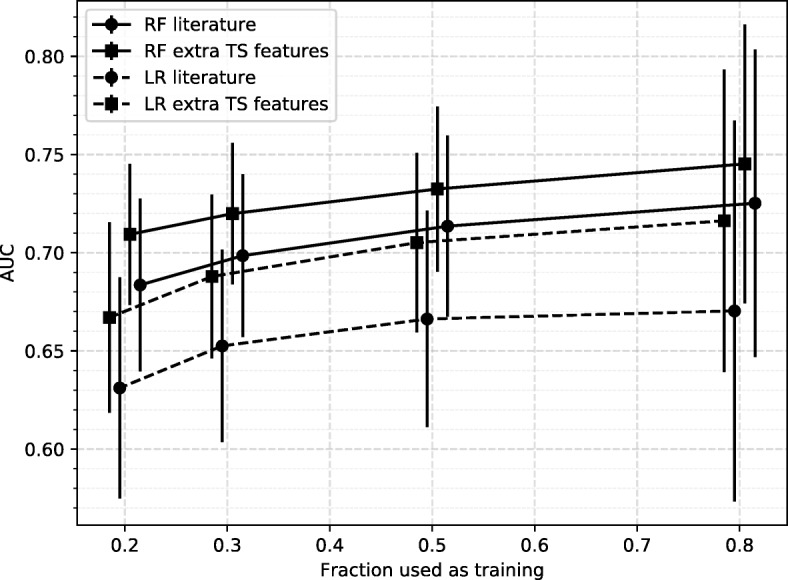
Results of the disability progression task Results are shown for different sizes of training set. Each point represents an average over 100 000 test sets, with the error bar indicating the standard deviation. Results are shown for the baseline model which uses a subset of known features (Latency, EDSS at *T*_0_ and age), as well as a model where we add additional TS features. Abbreviations used: RF Random Forest, LR Logistic Regression, TS Time series. These results are represented numerically in Table 2

The main results are shown graphically in Fig. 4, and numerically in Table 2. As is to be expected, we see that the overall performance of the pipeline increases as the size of the training set increases, while the variance of the result also increases due to the smaller size of the test set. The general trend we see is that adding the extra time series features improves the performance on the independent test set, but only marginally. RF performs better than LR both with and without the additional TS features, with the difference being especially evident when not adding them. The figure indicates that increasing the dataset size further would improve the performance.

**Table 2:** Results of the disability progression task

	RF			LR		
% train	Lit	TS	Sign	Lit	TS	Sign
80	0.73±0.08	0.75±0.07	12.1%	0.67±0.10	0.72±0.08	21.8%
50	0.71±0.05	0.73±0.04	18.7%	0.67±0.06	0.71±0.05	35.8%
30	0.70±0.04	0.72±0.04	24.7%	0.65±0.05	0.69±0.04	37.9%
20	0.68±0.04	0.71±0.04	30.1%	0.63±0.06	0.67±0.05	42.2%

The leftmost column indicates what percentage of the dataset was used for training. Results are shown for the classifier using just latencies, EDSS at *T*_0_ and age (Lit), and for the classifier trained on these features + additional TS features (TS). RF (Random Forest) and LR (Logistic Regression) indicate the classifier that was used. The *sign* column indicates the percentage of splits with a significant improvement, according to the DeLong test. These results are shown graphically in Fig. 4. The values after ± indicate the standard deviations

As a check for our assumption of using only a subset of the literature features, we also checked the performance when adding additional literature features to the classifier (peak-to-peak amplitude, gender and type of MS). The resulting model performed worse than the model using just 4 literature features in almost every case, and in the cases where it does increase it does so by a negligible margin. It also degrades the performance gain by adding TS features, presumably due to overfitting. This reaffirms our decision of using just the latencies, the age and the EDSS at *T*_0_.

### Significance test of performance increase

**Fig. 5:**
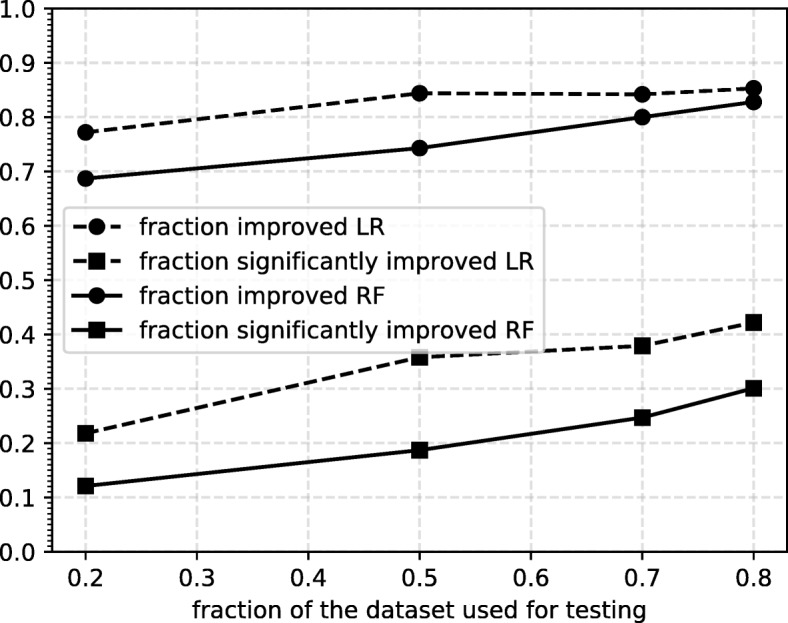
Significance results The results of the DeLong test on the improvement by adding TS features. We show both the fraction of splits that show an improvement and the fraction of splits that show significant improvement according to the DeLong test

**Fig. 6:**
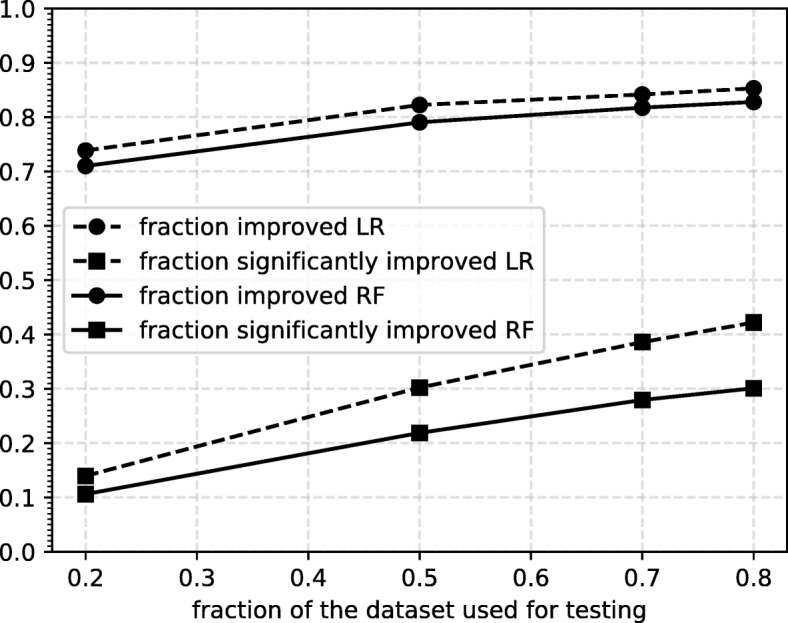
Significance results single model Results of the significance tests, using a single model (trained on 20% of the dataset), and tested on various sizes of test set. Both the fraction of improved splits and the fraction of significantly improved splits are shown. The trend suggests more data would likely increase the fraction of splits that show significant improvement

To check whether the increase in performance by adding TS features is significant, we employ the DeLong test [57] which tests the hypothesis that the true difference in AUC of the model with and without TS features is greater than zero. For each split we compare the ROC curves of the classifier with and without the additional TS features. The results are shown in Fig. 5. We observe that the percentage of splits with significantly improved performance increases with the size of the testset, reaching a maximum at 80% of the dataset used for the testset. We argue that the low fraction of significant improvement is mainly due to the power of the test. To support this further we show the significance percentages for a single model (the one trained on 20% of the dataset), tested on subsets of the remaining 80% of increasing size. The results are shown in Fig. 6, from which we see the fraction of significant splits increases steadily with the number of samples in the test set.

### Selected features

It is interesting to see which TS features are often found to be important according to our feature selection method. As the pipeline is run independently for 1000 times we have 1000 ranked sets of features deemed important by the feature selection. We consider only the train/test-split where the training set consists of 80% of the dataset, as the feature selection is most stable in this case. We consider the anatomies separately as the selected TS features are different for each. Here we give only a brief overview of the features that we found to be most important. For a ranked list of the 20 most important features for both APB and AH we refer the reader to the additional files [see Additional file 1]. There we also provide a way of obtaining the code used to generate these features.

**APB:** The feature most often found to be important ranks in the final 10 features for 83.9% of splits. In 74.7% of splits it ranks in the top 3. The feature in question is calculated by sliding a window of half the length of the TS across the TS in steps of 25% of the TS (so a total of 3 windows is considered). For each window, the mean is calculated. Finally, the standard deviation of these means, divided by the standard deviation of the entire TS, is calculated. In practice this feature seems to characterize how fast the TS returns to an average of zero after the initial peak. The other high-ranking features are mostly other sliding window calculations or features that compute characteristics of the spectral power of the TS. The prominence of these features drops off quickly, e.g., the second highest ranking feature occurs in the top 4 for 39% of splits.

**AH:** For AH one feature in particular stands out. It is included in the final 10 features for 97.5% of splits. In 90.6% of the splits, it is in the top 3 most important features. In fact, it is very consistently marked as being important for various methods of feature selection (cfr. Additional file 1), more consistently even than all the other literature features (latency, EDSS and age). Using just this feature extracted from the AH measurements, a prediction model can achieve a performance of 0.7±0.07 AUC (mean and standard deviation). This makes it a very interesting candidate for further research. Unfortunately, it is not very interpretable. The feature is calculated by fitting an autoregressive model to the timeseries, and evaluating its performance on 25 uniformly selected subsets of the timeseries of 10% the total length of the time series. The evaluation is based on 1-step ahead prediction. The difference between the real and predicted value forms a new TS, of length 192 in our case. The autocorrelation at lag 1 is calculated of each of these 25 TS. Finally, we take the absolute value of the mean of these 25 autocorrelation values. Further research could be done to determine why this particular feature is found to be this important. Other high-ranking features include those that quantify the level of surprise of a data point, given its recent memory. The remaining features show no clear pattern of type. As was the case for APB, we find that these lower ranked features’ prominence drops off rapidly.

**Fig. 7:**
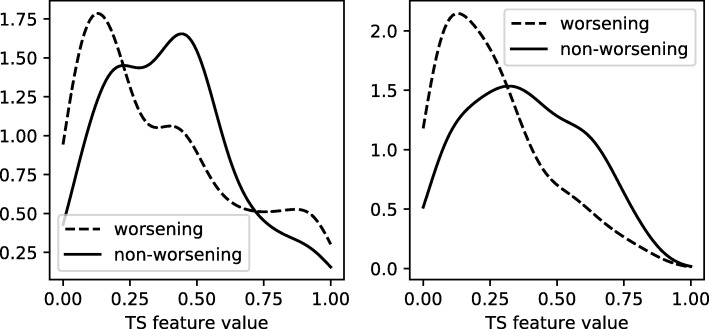
TS feature distributions The distributions of the most important additional TS feature for APB (left) and AH (right). The dashed lines represent the distributions of the TS feature of patients that show progression after 2 years, whereas the solid lines are for those that do not progress. The distributions are normalized separately

The distributions for the most important features for each anatomy are shown in Fig. 7. These figures were generated using kernel density estimation with a Gaussian kernel. Despite significant overlap in the distributions for the two classes, there is a definite difference between the two. For APB the distributions suggest that patients that are going to worsen have a more rapid return to an average of zero after the initial peak than patients that will not worsen. For AH such an intuitive interpretation is difficult due to the oblique nature of its most important TS feature. The features that were found have a few hyperparameters associated with them that could be optimized to further boost the performance of the classifier. We will not be doing this here, as these features were selected by looking at all splits at once, which covers the complete dataset. Their performance should be evaluated on another independent test set.

## Discussion

This paper presents the first analysis on a new dataset, containing the full time series of several EP types. The idea was to extract a large number of features from the MEP from different time series analysis methods, and use a machine learning approach to see which ones are relevant. Improving the prediction of disability progression compared to using only the latencies, age, and EDSS at *T*_0_ was quite difficult, despite its larger size compared with the literature. The main problem was overfitting. We expect the algorithm to deal quite well with noisy input features, caused by generic measurement noise and variations in the manual latency annotation. In fact, small input noise could help to avoid overfitting. The main reason for overfitting seems, to us, the noise in the binary target, caused mainly by an inherent unreliability of the EDSS measurement itself, and the lack of confirmation of the disability progression. Furthermore, there is an unknown upper limit for this task: even with an infinitely large cohort, the task will not be perfectly solvable with the biomarkers in our model. Our results shown in Fig. 4 suggest that this upper limit is not yet reached, although we could be close to it.

On average, one in four TS features that remained after the mutual information and hierarchical clustering steps was found to contain at least some information relevant to the prediction task, though only a small subset contained a strong enough signal to be consistently marked important across multiple train/test splits. Nevertheless, a significant improvement was found by adding extra features that showed high importance. The usefulness of non-linear methods is also clearly demonstrated.

Much more remains to be investigated on this dataset. Given the large amount of literature on the usefulness of mmEP (as discussed in the introduction), the largest performance improvement is most likely achieved by including the VEP and SEP. Given the large differences in measurement times and frequencies of the different EP modalities, one has to decide between throwing away a lot of data, or using more elaborate techniques that robustly handle missing data. The second option that has potential for significant improvement is analyzing the whole longitudinal trajectory of the patient [58]. This in contrast to our current analysis, where a single visit is used for predicting progression over 2 years. Inclusion of all (sparsely measured) mmEP and longitudinal modeling can be combined, and is an active research area [59, 60]. An obvious extension is to use TS algorithms not included in HCTSA. For example, another library with qualitatively different TS analysis methods is HIVE-COTE [49].

We have constricted ourselves to predicting progression over 2 years. This choice was made because it frequently occurs in the literature, and it leads to many training samples. Longer or shorter time differences are also of interest. It is, furthermore, believed by some clinicians that EPTS pick up disease progression faster than EDSS. One could check this by using short time-scale EPTS changes (e.g., 6 months) to predict EDSS changes on longer time-scales [23], or to detect non-response to treatment.

The obvious left-right-symmetry of the limb measurements is taken into account in a rudimentary way. Incorporating this symmetry in a more advanced way could boost performance. Data augmentation can be used to expand the size of the training set, which could stabilize the performance estimate. We note that even small neural networks are difficult to train on the current dataset. Data augmentation could make them competitive.

While the achieved AUC of 0.75±0.07 is impressive for a model with only MEP latency, EDSS at *T*_0_, age, and a few additional MEP TS features, there is surely an upper limit to what mmEP can predict. Other variables such as, e.g., MRI, cerebrospinal fluid, and genomic data could boost performance [5]. A very important variable that is currently not included is the type of DMT the patient is on. In the absence of a single, highly predictive marker, personalization will depend on a combinations of markers. Indeed, several studies show that a multi-parametric approach may improve our prognostic ability in MS [61, 62]. It involves the development of predictive models involving the integration of clinical and biological data with an understanding of the impact of disease on the lives of individual patients [63]. Besides the inclusion of extra biomarkers, another step of great practical importance is to move towards multi-center design studies. How well mmEP data from different centers can be combined remains an open and very important question [64, 65].

Our results contribute to the long-term goal of improving clinical care of people with MS in several ways. We add evidence to the hypothesis that EP are a valuable biomarker in personalized prediction models. This is important, because the precise value of EPs for monitoring MS is still under discussion [35–37]. Our evidence is stronger compared to previous work, because the performance is tested on an independent test set, and the EPs were measured in routine clinical follow-up.

If a model predicts that the patient is likely to progress in disability, this could be a sign of treatment inefficacy, and a switch to a different or more aggressive DMT could be done. Improving the performance of predictive models can therefore lead to a faster optimal treatment choice, and result in slower disability progression.

Finally, if our identified extra features are confirmed in larger, multi-center studies, their evolution over time can be used to give additional feedback to the caregivers on the disease evolution.

## Conclusions

Multiple sclerosis is a chronic disease affecting millions of people worldwide. Gaining insight into its progression in patients is an important step in the process of gaining a better understanding of this condition. Evoked potential time series (EPTS) are one of the tools clinicians use to estimate progression. The prediction of disability progression from EPTS can be used to support a clinician’s decision making process regarding further treatment, and reduce uncertainty for patients about their disease course.

We presented a prediction model for disability progression after 2 years, trained on a dataset containing all available motor EP measurements from the Rehabilitation & MS Center in Overpelt, Belgium. Any patient with two-year follow-up is included. It is an order of magnitude larger than most datasets used in previous works, and for the first time includes the raw time series, as opposed to just the high-level features extracted from them (i.e. latencies, peak-to-peak amplitude, and dispersion pattern). The dataset consists of individuals undergoing treatment, which is clinically the most relevant scenario. We plan to make this dataset publicly available in the near future.

We found that adding additional features extracted from the raw time series improves performance, albeit marginally (*Δ*AUC =0.02 for the best performing classifier). Results suggest that the model would benefit from an increased dataset size. We found that linear models, often used in previous works, are significantly outperformed by the random forest classifier, especially when not adding extra TS features (*Δ*AUC =0.06). Given the limited number of biomarkers in the model (EDSS at *T*_0_, MEP, and age) and heterogeneity of the cohort, the reported performance (AUC 0.75±0.07) is quite good. We took an initial look at the features that were found to boost predictive power and found a few candidates that might be a good starting point for further research. The feature found to be important for the feet (AH) (see “Selected features” section) is particularly robust to all feature selection methods (cfr. Additional file 1), even more so than the features currently considered by clinicians. If its importance is confirmed in larger, multi-center studies, further investigation into what this feature measures could potentially lead to new physiological insights, and could guide clinicians in their interpretation of the measurements.

## Supplementary information

**Additional file 1** Most important TS features / alternative feature preselection algorithms. This file contains tables of the 20 most prominent TS features across the 1000 train/test splits, for both APB and AH anatomies. It also provides a way of obtaining the code used to generate these features. There is also a section on a few alternatives for the feature preselection step. Lastly the process of chosing hyperparameters is discussed and motivated.

## Data Availability

The dataset analyzed during the current study is currently not publicly available due to privacy concerns but is available from the corresponding author on reasonable request. We aim to release the dataset as well as the code used to generate the results publicly after taking steps to ensure the full anonymity of the patients in accordance with local data protection laws.

## Abbreviations

A list of the abbreviations used throughout this work: AH: Abductor Hallucis
AIC: Akaike information criterion
APB: Abductor Pollicis Brevis
AUC: Area under curve (of ROC, see below)
BAEP: Brainstem auditory EP (EP see below)
BIC: Bayesian information criterion
EDSS: Expanded disability status scale
EP: Evoked potentials
EPS: EP score
EPTS: EP time series
FWO: Fonds Wetenschappelijk Onderzoek
HCTSA: Highly comparative time-series analysis
LR: Logistic regression
MEP: Motor EP
mmEP: Multimodal EP
MRI: Magnetic resonance imaging
MS: Multiple sclerosis
MSE: Mean-squared error
PPMS: Primary progressive MS
RF: Random forest
ROC: Receiver operating characteristic
SD: Standard deviation
SEP: Somatosensory EP
SPMS: Secondary progressive MS
TS: Time series
VEP: Visual EP
