# Supplementary material for: Machine learning analysis of motor evoked potential time series to predict disability progression in multiple sclerosis
Source: BMC Neurol. 2020 Mar 21;20:105. doi: 10.1186/s12883-020-01672-w (PMC7085864; doi:10.1186/s12883-020-01672-w)
Supplement: Supplementary file 1 — Additional file 1 Most important TS features / alternative feature preselection algorithms. This file contains tables of the 20 most prominent TS features across the 1000 train/test splits, for both APB and AH anatomies. It also provides a way of obtaining the code used to generate these features. There is also a section on a few alternatives for the feature preselection step. Lastly the process of chosing hyperparameters is discussed and motivated. [file 12883_2020_1672_MOESM1_ESM.pdf]

Machine Learning Analysis of Motor Evoked Potential Time Series to Predict Disability Progression in Multiple Sclerosis: Additional file 1

Jan Yperman, Thijs Becker, Dirk Valkenborg, Veronica Popescu, Niels Hellings, Bart Van Wijmeersch, and Liesbet Peeters

1 Highest ranking features

We provide the top 20 most important features for the disability progression task, both for APB (Table 1) and AH (Table 2). Their ranking is derived as follows: For each split we have 10 ranked features. For each unique feature that occurs across the splits we assign a score from 0 to 9 based on its position in a particular split (0 for being in first place, 9 for being in 10th). If it does not occur in a split it receives a score of 10. We add the scores of each of these features for all splits. The feature with the lowest score will be considered the most important. This score is included in the tables in the column *score*.

In the tables we also included the HCTSA ID, which can be used to get a description of the feature, as well as retrieve the code used to generate this feature. Instructions on how to do this can be found online[1].

The remaining columns are the name of the feature (as generated by HCTSA) and the percentage of splits where the feature occurs in the top n.

2 Alternative feature selection

In the manuscript we outlined a data analysis pipeline which includes a couple of initial steps that cut down on the number of features from roughly 5000 to a couple hundred (See Figure 2 in the main text). We achieve this by retaining only the top 10% best features, based on their mutual information with the target. Features that are highly correlated with one another are then discarded using hierarchical clustering based on the correlation distance. This step could have a great impact on the end result, so we check how other feature reduction techniques affect the performance.

To address this issue, we ran the complete pipeline again, this time using some other frequently employed techniques to replace both the mutual information step and the hierarchical clustering step. We opted for an ANOVA F-value to replace the mutual information, and a greedy feature selection based on the Pearson correlation to replace the hierarchical clustering. The Pearson correlation and the correlation distance are closely related, but the way of clustering the features differs: For hierarchical clustering we build a rooted tree (dendrogram) based on the correlation distance using the UPGMA algorithm, which we subsequently flatten using a cophenetic distance cutoff of 0.1. For the greedy feature selection based on the Pearson correlation, on the other hand, we calculate the correlation matrix between the features and start removing features (keeping one of each pair) starting from the most highly correlated features and continuing until there are no more pairs of features that are correlated more than 0.9. We ran the pipeline again for the case where 80% of the data set is used for training as this would lead to the most stable feature selection. The results are shown in Table 3.

[1]https://hctsa-users.gitbook.io/hctsa-manual/analyzing_visualizing/interpreting-features

name	hctsa id	top 1	top 3	top 5	top 10	score
SY SlidingWindow m s2 2	561	43.4%	74.7%	80.7%	83.9%	2414
SY LocalGlobal l500.absmean	762	3.8%	26.0%	50.5%	82.8%	5027
CO AddNoise 1 gaussian.ami at 5	1264	2.4%	24.1%	50.5%	79.4%	5174
SY SlidingWindow m s 4 1	554	4.4%	26.7%	42.8%	59.6%	6062
SP Summaries pgram hamm.fpoly2 sse	4307	14.5%	38.2%	43.4%	45.3%	6078
PP Compare poly2.swms10 1	5795	3.6%	17.2%	32.5%	51.4%	6804
PP Compare spline44.statav4	5912	15.9%	27.0%	30.7%	33.3%	7128
PP Compare spline24.statav6	5882	0.3%	5.8%	15.8%	43.6%	8008
SP Summaries pgram hamm.peakPower 2	4271	0.9%	4.3%	14.1%	42.0%	8163
SY TISEAN nstat z 4 1 3.mean	4778	4.8%	12.1%	16.0%	19.5%	8507
SY SlidingWindow s ent2 10	597	0.1%	0.8%	5.3%	31.0%	8915
SP Summaries pgram hamm.fpoly2csS p1	4304	0.3%	2.6%	8.6%	19.6%	9049
PP Compare spline64.statav4	5943	0.8%	5.2%	8.5%	14.1%	9127
CO Embed2 tau.eucds1	1928	0.1%	0.9%	4.0%	20.5%	9259
SY TISEAN nstat z 4 1 3.std	4783	0.2%	2.3%	5.8%	15.6%	9272
FC Surprise T1 100 5 udq 500.uq	2405	0.1%	2.1%	6.2%	13.1%	9316
SP Summaries pgram hamm.fpoly2csS p2	4305	0.0%	1.3%	4.6%	15.7%	9325
PP Compare medianf3.swms2 2	6040	0.1%	1.9%	5.5%	14.1%	9337
MF AR arcov 4.res AC1	3861	0.0%	0.5%	3.1%	19.0%	9340
SY SpreadRandomLocal 100 100.stdstd	2136	0.0%	1.0%	4.4%	16.5%	9353

Table 1: The 20 most important features across all 1000 train/test splits for APB

Looking at the cause for the performance drop, we can see that in the case of the F value, the best **APB feature** is almost always discarded, most likely due to the fact that the F value is only sensitive to linear dependencies[2]. When using mutual information however, it is picked up in 85% of the splits. For the AH feature on the other hand, it does not get discarded by either mutual information or the F value, indicating there is a linear dependency between it and the target.

The **AH feature** may, however, get discarded during the removal of highly correlated features. Looking into this more closely it turns out this happens due to the fact that the best AH feature is highly correlated with a feature that does not perform quite as well. The features in question are:

- *MF_CompareTestSets_y_ar_best_uniform_25_01_1.ac1s_mean*
- *MF_CompareTestSets_y_ar_best_uniform_25_01_1.ac1s_median*

where the median feature performs worse for our prediction task. The difference between the two features is only minor, causing them to be highly correlated. Due to their correlation they end up in the same cluster, at which point it depends on which feature is chosen from each cluster. In our case, we chose the first occurring feature for convenience, which turned out beneficial as this is the best performing feature of the two. This is not the case, however, when using the Pearson correlation matrix, causing the best feature to be dropped during this step. This results in a performance drop to 0.738. It's interesting to note that in absence of the *mean* feature, the *median* feature is selected to be the most informative by Boruta.

To verify that we haven't missed any important features due to a similar process, we ran the pipeline again, this time removing the highly correlated feature filter altogether. This results in the same features being found as the original pipeline, with the same performance.

Another popular feature selection technique is the Fast Correlation Based Filter (FCBF), first proposed by Yu and Liu [2003]. We will now compare its performance with that of the pipeline outlined in the main

[2]This is illustrated nicely at `https://scikit-learn.org/stable/auto_examples/feature_selection/plot_f_test_vs_mi.html`

name	hctsa id	top 1	top 3	top 5	top 10	score
MF CompareTestSets y ar best uniform 25 01 1.ac1s mean	7062	69.3%	90.6%	94.4%	97.5%	861
SB BinaryStats iqr.longstretch0	3490	5.8%	36.8%	51.2%	61.3%	5420
PH ForcePotential sine 1 1 1.median	1655	1.6%	14.9%	31.9%	53.7%	6886
FC Surprise T2 20 2 q 500.std	2310	0.7%	17.9%	31.5%	47.3%	7106
FC Surprise dist 20 2 q 500.mean	2242	0.4%	8.9%	25.0%	52.2%	7297
EN Randomize statdist.statav5diff	2854	4.3%	19.1%	27.9%	38.2%	7357
MF CompareTestSets y ar 4 rand 25 01 1.ac1s mean	7042	1.5%	16.5%	28.5%	39.9%	7394
WL DetailCoeffs db3 max.max1on2 median	6526	0.9%	10.5%	21.1%	31.3%	8052
FC Surprise dist 50 3 q 500.mean	2250	0.0%	1.8%	8.8%	31.1%	8704
FC Surprise dist 50 3 q 500.std	2254	0.2%	3.9%	8.8%	23.4%	8927
MF FitSubsegments arsbc uniform 25 01.sbcs range	6981	0.0%	3.8%	7.8%	22.8%	8980
EN Randomize statdist.statav5hp	2855	2.5%	8.0%	10.6%	13.7%	8989
EN Randomize permute.statav5fexpr2	2978	0.2%	2.4%	6.0%	21.4%	9050
MF GP hyperparameters covSEiso covNoise 1 50 random i.meanS	6396	1.1%	5.0%	8.7%	12.4%	9160
MF GP hyperparameters covSEiso covNoise 1 50 random i.mlikelihood	6389	0.8%	6.0%	8.4%	10.6%	9261
FC LocalSimple mean3.tauresrat	3017	3.7%	6.6%	7.0%	7.4%	9334
SP Summaries pgram hamm.linfitsemilog all a2	4340	0.2%	3.9%	6.2%	10.4%	9376
StatAvl500	550	0.2%	1.6%	4.9%	12.5%	9430
SP Summaries fft logdev.linfitsemilog all a1	4587	0.0%	1.7%	4.6%	12.1%	9452
FC LocalSimple lfittau.tauresrat	3137	0.1%	1.7%	4.7%	10.7%	9481

Table 2: The 20 most important features across all 1000 train/test splits for AH

Feature selection	Performance
Mutual information + Hierarchical clustering	$\mathbf{0.745 \pm 0.071}$
Mutual information + Greedy selection w. Pearson correlation	0.738 ± 0.072
F value + Hierarchical clustering	0.738 ± 0.073
F value + Greedy selection w. Pearson correlation	0.733 ± 0.073
FCBF	0.738 ± 0.07

Table 3: Results for the alternative preselection steps, with standard deviations. Except for FCBF, all pipelines include the Boruta step. The values after '±' indicate the standard deviation.

manuscript, i.e., the mutual information filtering followed by hierarchical clustering on the correlation distance and finally the Boruta feature selection algorithm. The main advantage of the FCBF is its speed, running in mere seconds whereas the Boruta pipeline takes well over an hour on a single core. However, we find that performance-wise, the Boruta pipeline is superior for our task.

Running the pipeline with the FCBF feature selection, we obtain a performance of $\mathbf{0.738 \pm 0.07}$ (mean and standard deviation), to be compared with $\mathbf{0.745 \pm 0.07}$ obtained by the Boruta pipeline, also shown in Table 3. In Table 5 and 6 we show the top ranked features selected by the FCBF algorithm for APB (arms) and AH (legs) respectively, similar to those shown in Section 1. Looking at these tables, there is certainly an overlap in the chosen features. For the AH features, the best feature, as determined by the Boruta algorithm, is also recovered using FCBF. However, the best feature for APB is not recovered, which most likely explains the difference in performance on the test set.

We also find that the FCBF algorithm seems to be less sensitive to feature importance. We illustrate this in Table 4. There we show how often the literature features (latency, EDSS at T_0 and age) are labeled as important by both algorithms. These features have been shown to be relevant by various works in the literature, yet it seems FCBF marks them as important far less consistently than the Boruta algorithm. This makes the fact that FCBF recovers the best AH feature in 83% of splits rather impressive.

	FCBF	Mutual inf + correlation + Boruta
Latency	3.38%	74.80%
EDSS T_0	6.75%	83.80%
Age	7.65%	47.10%

Table 4: Comparison of the recovery of known literature features by both feature selection algorithms. The percentage of splits is shown where the feature in question was labeled to be important.

3 Hyperparameter choice

In this section we will further discuss the choice of hyperparameters for the model. In particular, the following parameters were set:

- **maximum # features to be passed to the classifier [N_FEAT_RETAIN]:** As discussed in the main text we use a validation set to determine how many of the top-n features (as ranked by the boruta alogorithm) are passed to the model. We set an upper limit to this number as for some splits the validation scores may be somewhat noisy, leading to a high number of features being passed to the classifier. For our final results we set this value to 6.
- **# trees in the random forest [N_EST]:** This value is a trade-off between classifier performance and computational intensity. We set this to 100 for our final results.

name	hctsa id	top 1	top 3	top 5	top 10	score
EN Randomize permute.ac3fexpb	561	21.0%	26.2%	26.2%	26.2%	7438
SP Summaries pgram hamm.fpoly2 rmse	762	22.0%	22.0%	22.0%	22.0%	7800
PH ForcePotential sine 1 1 1.ac1	1264	2.7%	20.3%	20.3%	20.3%	8197
EN Randomize dyndist.sampen2 015fexpr2	554	0.0%	9.4%	23.5%	25.2%	8226
PH Walker runningvar 15 50.sw maxrat	4307	0.0%	9.2%	22.2%	23.7%	8308
SB TransitionpAlphabet 20 ac.trfexp a	5795	4.2%	16.4%	16.4%	16.4%	8488
MD rawHRVmeas.trisqrt	5912	9.8%	11.8%	11.8%	11.8%	8840
MD rawHRVmeas.SD1	5882	4.5%	12.1%	12.1%	12.1%	8876
PH Walker biasprop 05 01.w ac2	4271	8.2%	9.1%	9.1%	9.1%	9099
MD rawHRVmeas.SD2	4778	0.0%	1.6%	11.8%	13.4%	9124
TSTL delaytime 01 1.tau1	597	0.0%	7.2%	8.4%	8.4%	9286
WL dwtcoeff sym2 5.mind l3	4304	0.4%	7.8%	7.9%	7.9%	9319
DN RemovePoints max 01.ac3rat	5943	1.7%	7.0%	7.0%	7.0%	9356
MF arfit 1 8 sbc.maxImS	1928	0.0%	2.3%	8.4%	9.6%	9360
DN RemovePoints max 01.ac3diff	4783	1.0%	6.8%	6.8%	6.8%	9382
EN Randomize statdist.ac2fexpb	2405	4.7%	5.8%	5.8%	5.8%	9431
WL coeffs db3 4.wb50m	4305	0.0%	0.5%	6.1%	8.7%	9474
SP Summaries welch rect.numPeaks	6040	0.0%	1.5%	6.3%	7.2%	9516
PP Compare poly2.statav4	3861	0.0%	2.5%	5.3%	5.4%	9602
CO Embed2 tau.eucds3	2136	3.3%	3.9%	3.9%	3.9%	9616

Table 5: The 20 most important features across all 1000 train/test splits for APB, for the FCBF algorithm

name	hctsa id	top 1	top 3	top 5	top 10	score
MF CompareTestSets y ar best uniform 25 01 1.ac1s mean	7062	83.3%	83.3%	83.3%	83.3%	1670
MF GP hyperparameters covSEiso covPeriodic covNoise 1 200 resample.meanS	3490	0.0%	2.5%	37.7%	45.4%	7121
MF StateSpace n4sid 1 05 1.acmnd0	1655	0.0%	25.1%	30.8%	30.8%	7476
EN Randomize statdist.statav5fexpr2	2310	0.0%	24.0%	24.4%	24.4%	7845
PP Compare rav4.olbt s5	2242	0.0%	14.8%	26.2%	27.7%	7942
MF armax 2 2 05 1.ac2	2854	0.0%	16.0%	21.7%	21.8%	8226
SB TransitionMatrix 2ac.T1	7042	0.0%	2.7%	18.4%	24.4%	8475
MF AR arcov 5.a5	6526	0.0%	10.5%	14.5%	14.6%	8857
EN Randomize dyndist.statav5fexpr2	2250	0.0%	10.8%	11.4%	11.4%	9031
CO AddNoise 1 gaussian.ami at 20	2254	0.0%	7.0%	12.3%	12.5%	9058
CO AddNoise 1 gaussian.ami at 10	6981	0.0%	6.9%	11.0%	11.1%	9140
PP Compare spline44.swss10 1	2855	0.0%	5.9%	9.2%	9.4%	9290
MF armax 2 2 05 1.acmnd0	2978	0.0%	7.2%	7.2%	7.2%	9368
MF armax 1 1 05 1.p4 5	6396	0.0%	6.6%	7.7%	7.7%	9370
SY SpreadRandomLocal 200 100.stdtaul	6389	0.5%	6.8%	6.9%	6.9%	9384
SY DriftingMeann5.mean	3017	0.0%	1.0%	5.8%	7.3%	9528
MF StateSpace n4sid 1 05 1.ftbth	4340	0.0%	1.6%	5.7%	6.0%	9579
NL DVV 3 100 2 50 10 default.meanDiffSurr	550	0.0%	0.1%	3.4%	6.1%	9648
MF GP hyperparameters covSEiso covPeriodic covNoise 1 200 resample.maxS	4587	0.0%	0.4%	4.3%	5.2%	9671
PH Walker momentum 5.sw ac1rat	3137	0.0%	3.5%	3.7%	3.7%	9675

Table 6: The 20 most important features across all 1000 train/test splits for AH, for the FCBF algorithm

Feature	% important
Type of MS	0.05
Gender	0
PPA L	4.60
PPA R	16.55

Table 7: Percentage of splits for which additional metadata is marked as being important by the feature selection pipeline, for APB and AH together.

- **minimum samples split [MIN_SAMPLES_SPLIT]:** This value determines how many samples are required to warrant the split of an internal node of the random forest, and is used prune the forest and subsequently avoid overfitting. For our final results we set this value to 0.1 (10% of the total number of samples).
- **literature features [LIT_FEAT]:** Our choice of which features from the literature to use. For our final result we used the latency of the left and right limb, the EDSS at T_0 and the age.

To avoid having different hyperparameters for each of the splits, we set these values to the same value for all splits. These global parameters were chosen using trial and error based on the average of the cross-validation scores (4-fold grouped k-fold) on the training sets of all the splits. However, as this average score covers the entire dataset, this could still lead to overfitting. To minimize this risk, only a few hyperparameter values were evaluated this way while picking a roughly functional value for each of the hyperparameters. To assess the likelihood of these choices having led to an overfit we plot the performance of the model for a range of values of these hyperparameters in Figure 1. It is clear the graph that the model performance is robust to different values of the hyperparameters, bar any extreme values. For the N_FEAT_RETAIN feature and the N_EST feature the final chosen value was not actually the best choice for this dataset, but the difference is negligible.

We also ran the model again while choosing the MIN_SAMPLES_SPLIT value based on 4-fold cross-validation on the training set for each split individually (as opposed to choosing one global value which is set across all splits, which is what we did for the main results). The possible values were 0.01, 0.05, 0.1, 0.15 and 0.2. The model then performs slightly worse (0.740 as opposed to 0.745). Similarly, when doing this with the model that contains only the literature features, we see a performance drop from 0.725 to 0.716. Therefore we see that adding the additional time series features to the model increases the performance when we use cross-validation to determine the hyperparameters for each split individually, in line with the main results. We opted not to do this for the main results as discussing the hyperparameters would have become intractable, since each split would have a different set of hyperparameters.

For the LIT_FEAT hyperparameter we selected the latencies, the EDSS at T_0 and the age based on the fact that they were marked as being important in the literature. As an indication of their importance, we show in how many splits these are marked as important by the feature selection pipeline in Table 7. A few other extra features were added to the feature selection pipeline along with the timeseries features. Comparing these results to those of the literature features we did include, as shown in Table 4, it's clear that these values can be safely discarded. It should be noted that the type of MS variable has a lot of missing data, and while the missing values were inferred from various other metadata, it may not be sufficiently accurate.

References

L. Yu and H. Liu. Feature selection for high-dimensional data: A fast correlation-based filter solution. In *Proceedings of the 20th international conference on machine learning (ICML-03)*, pages 856–863, 2003.

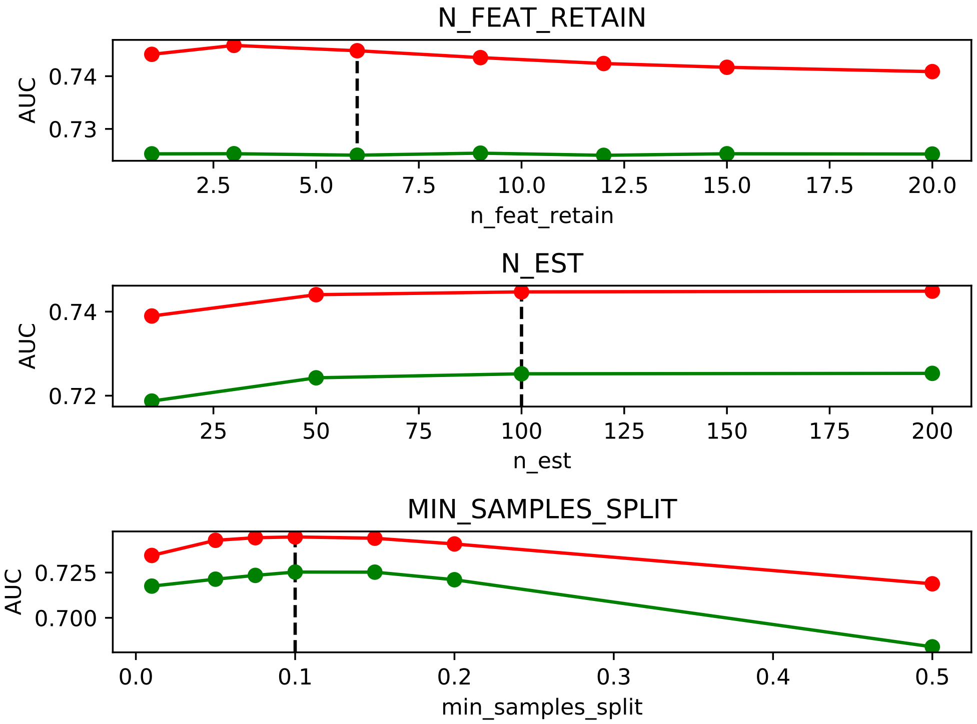

Figure 1: The effect of various hyperparameters on the performance of the model, for the model with (red) or without (green) extra EPTS features. The hyperparameters used for the final model are shown by vertical, dashed lines.
